# Impact of a 3-year multi-centre community-based intervention on risk factors for chronic disease and obesity among free-living adults: the Healthy Alberta Communities study

**DOI:** 10.1186/s12889-016-3021-1

**Published:** 2016-04-18

**Authors:** Ellina Lytvyak, Dana Lee Olstad, Donald P. Schopflocher, Ronald C. Plotnikoff, Kate E. Storey, Candace I. J. Nykiforuk, Kim D. Raine

**Affiliations:** School of Public Health, University of Alberta, 3-300 ECHA, 11405 87 Ave, Edmonton, AB T6G 1C9 Canada; Institute for Physical Activity and Nutrition, Deakin University, 221 Burwood Highway, Burwood, VIC 3125 Australia; Priority Research Centre in Physical Activity and Nutrition, The University of Newcastle Callaghan, Callaghan, NSW 2308 Australia

**Keywords:** Community-based intervention, Systolic blood pressure, Diastolic blood pressure, Blood pressure, Hypertension prevention, Obesity, Anthropometric measures, Chronic disease prevention

## Abstract

**Background:**

Healthy Alberta Communities (HAC) was a 3-year community-based intervention to reduce lifestyle-related risk factors for chronic disease and obesity at a population-level. The current paper examines changes in blood pressure (BP) and anthropometric indicators within HAC communities compared to secular trends.

**Methods:**

Between 2006 and 2009, this community-academic partnership sought to create environments supportive of healthier dietary and physical activity behaviours within four diverse communities in Alberta, Canada. Height, weight, waist and hip circumference and BP were measured among 1554 and 1808 community residents at baseline (2006) and follow-up (2009), respectively. A comparison sample was drawn from a representative national survey. Samples were stratified by age and change between pre- and post-intervention was assessed using t-tests. Changes in parameters over time between groups were compared using meta-analysis. The net difference in change in outcomes (change in intervention communities minus change in comparison group) represented the effect of the intervention.

**Results:**

Adjusted systolic (SBP) and diastolic (DBP) BP declined within most age groups in HAC communities from pre- to post-intervention. The net decline in SBP was 1 mmHg in 20–39 year olds (*p =* 0.006) and 2 mmHg in 40–59 year olds (*p =* 0.001), while the net decline in DBP was 3 mmHg in 20–39 year olds (*p <* 0.001), 2 mmHg in 40–59 year olds (*p <* 0.001) and 3 mmHg in 60–79 year olds (*p <* 0.001). The net increase in the proportion of individuals with normal BP was 5.9 % (*p <* 0.001), while the net decline in the proportion of individuals with stage 1 hypertension was 4.5 % (*p <* 0.001). BMI and body weight were unchanged. There was a significant net increase in waist and hip circumference among 20–39 year olds within intervention communities.

**Conclusions:**

Findings suggest HAC succeeded in shifting the population distribution of BP in a leftward direction. By contrast, anthropometric parameters remained unchanged or worsened within intervention communities. Therefore, while improvements in some clinical risk factors can be achieved through relatively diffuse and shorter-term community-level environmental changes, improvements in others may require interventions of greater intensity and duration. Evaluating the success of community-based interventions based on their efficacy in changing individual-level clinical indicators may, however, underestimate their potential.

## Background

Chronic diseases are the most important cause of morbidity and mortality worldwide. The major chronic diseases—cancer, cardiovascular disease, diabetes and chronic pulmonary disease now account for 63 % of all annual global deaths [1]. Similarly in Canada, cardiovascular diseases and cancer represent approximately 58 % of annual deaths [2]. Of the major chronic diseases, cardiovascular diseases, including conditions such as ischaemic heart disease, coronary heart disease, stroke, congestive heart failure, and end-stage renal disease, are the most prevalent and are on the rise [3, 4]. High blood pressure (BP), both systolic (SBP) and diastolic (DBP), has been repeatedly identified as the most universal strong, consistent, continuous, independent, and etiologically relevant contributor to cardiovascular diseases [5–8], and is responsible for 7 % of global disability-adjusted life years lost [9].

Obesity increases the risk of chronic disease, and indeed rates of obesity and chronic disease have risen in tandem over the past several decades [1, 4, 10]. In 2008, more than half a billion adults were obese worldwide [1], including 27.2 % of Canadian adults [11]. Chronic diseases and obesity are linked by two major shared, lifestyle-related preventable risk factors of unhealthy diets and insufficient physical activity [1, 3]. The disease burden attributable to unhealthy diets is particularly high [3]. In 2010, seven of the top 20 deaths and disabilities worldwide were related to poor diet [4], with excessive salt consumption and inadequate fruit and vegetable intake contributing 10 % of the total global burden of disease [9]. In Canada, it is estimated that 30,540 deaths could be averted annually if Canadians adhered to dietary recommendations [12]. Dietary factors, including low fruit, vegetable and potassium intakes, and excessive alcohol and sodium intakes are also major preventable causes of high BP, along with excess body weight and insufficient physical activity [13, 14].

Although the behavioural origins of chronic disease and obesity are clear, the drivers of these behaviours are varied and complex, and thus no nation has succeeded in reversing their high prevalence [15]. Factors at all levels interact to shape individual health behaviours, and therefore success in chronic disease and obesity prevention will require ecological approaches through multi-sectoral, multi-level, population-wide interventions [16]. Community-based interventions adopt an explicitly ecological approach to health promotion through integrated and comprehensive interventions targeting change among individuals, groups and community-level environments and policies [17, 18]. Their appeal stems largely from their potential to produce widespread change, as even if they are only modestly effective, small changes at a population-level can confer significant health benefits [19]. Community-based interventions are particularly appropriate to address chronic disease and obesity given that these conditions are so pervasive, affect all sociodemographic groups, share lifestyle-related risk factors that are shaped by environments and policies, and require sustained behaviour change for prevention [17].

The North Karelia Project in Finland stands out as among the most successful of community-based interventions. Through coordinated, multi-level policy and environmental change, the project is credited with achieving a 73 % reduction in age-adjusted coronary heart disease mortality from 1971 to 1995 [20], the benefits of which were still evident 35 years later [21]. Subsequent community-based health promotion initiatives patterned after the North Karelia approach have generally yielded mixed, and more modest improvements in population-level risk factors [18, 22]. A sizeable literature suggests that inadequate attention to contextual factors [23], methodological issues (eg. low statistical power, limitations of quasi-experimental designs, sampling issues), the influence of secular trends, smaller than expected effect sizes, limitations of the interventions (eg. insufficient duration and tailoring, low dose), absence of a robust theoretical underpinning, and insensitive evaluation tools may have contributed to disappointing results from some community-based interventions [18, 24, 25]. The collective learnings from the successes and failures of these studies suggest that best practice methods for community-based interventions include multi-sectoral partnerships, involving communities in program planning and implementation, tailored interventions, reduced access to unhealthy products, involvement of the non-health sector, addressing social inequalities in disease risk, coordinated multi-level interventions, rigorous process evaluation and a sufficient intervention dose [22, 26].

Healthy Alberta Communities (HAC) was a 3-year community-based intervention that sought to leverage lessons from past community-based interventions to expand the evidence base related to chronic disease and obesity prevention. The primary objective of this study was to assess the impact of a community-based intervention on risk factors for chronic disease and obesity. Summaries of the study’s conceptual framework [27] and overall findings (i.e. self-reported behavioural indicators, social conditions and objectively measured clinical outcomes) have been published [28]. Sense of belonging to community and objectively-assessed BP were the only measures in which improvements were observed in HAC communities relative to secular trends. The current paper examines changes in BP and anthropometric indicators according to age within HAC communities compared to secular trends. These more in-depth analyses were not reported in previous publications.

## Methods

### Context and intervention

The methodological details of the study have been previously described [27, 28]. Briefly, HAC was a community-academic partnership involving an intervention within four communities in Alberta, a geographically large, politically conservative, resource-rich province in Western Canada. The Government of Alberta selected the four communities to represent a range of demographic and geographic characteristics. Bonnyville and St. Paul were rural towns located in the northeastern part of the province, with economies predicated on agricultural and oil-field related activities. Norwood was a culturally diverse, socio-economically disadvantaged inner-city neighborhood located in Edmonton, Alberta’s capital city. Medicine Hat was a small, resource-rich city in the southeastern area of the province considered to be a service centre for the southern half of the province. Socio-demographic characteristics of the communities are summarized in Raine et al. [28].

Four Community Coordinators with established community networks were hired to link the research team with each community. During the initial years of the study, Community Coordinators built relationships with local stakeholders and worked with them to identify environmental determinants of chronic disease and obesity amenable to change at a community-level. Subsequent years focused on partnering with local individuals and organizations to intervene in the targeted areas. Key accomplishments included the expansion of community gardens, improved access to recreation and sport facilities, development of a healthy choice restaurant program, a program to provide subsidized local produce to food insecure households, and a linked trail system for active transportation [29].

### Data collection

#### Study design

Baseline data for all HAC communities were collected prior to intervention in spring, 2006, while follow-up data were collected at the conclusion of the study in spring, 2009. Data collection at both time points comprised three phases.

- Phase 1 was a cross-sectional telephone survey administered by an independent survey research firm. The survey took approximately 30 min to complete and assessed lifestyle behaviours (eg. diet, physical activity, smoking), health status (including height and weight) and cognitive change (intentions).
- Phase 2 involved physical measures of height, weight, waist and hip circumference and BP at a local measurement clinic. Participants were offered free transportation, light refreshments and a $20 CDN gift card to a local grocery store in exchange for participation. The clinics remained open 3–7 times per week (including evenings and weekends) over a 16-week period.
- Phase 3 was conducted exclusively in Medicine Hat, and consisted of blood collection by a phlebotomist to measure fasting glucose, total cholesterol, high- and low-density lipoprotein cholesterol levels and triglycerides. The third phase of the study is not discussed further in this paper as findings have been published [28].

#### Participants

Although other indicators were also considered relevant, the study was powered to detect a 1.5 % reduction in BMI. Random samples of adults proportionate to the number of inhabitants in each community were selected to participate in the telephone surveys in 2006 and 2009. In Norwood, Edmonton, participants were randomly selected from relevant postal code areas using the telephone directory. In the other three communities a Random Digit Dial sample frame was used to recruit participants using historical telephone lists. Individuals who participated in the survey were invited to attend the measurement clinic in their community to provide objective physical measures. Pregnant women and individuals in wheelchairs were excluded. The target and achieved enrollment numbers are summarized in Table 1.

**Table 1:** Self-reported characteristics of participants in intervention communities who completed physical measurements at baseline (2006) and follow-up (2009)

Parameters	Baseline *n =* 1554	Follow-up *n =* 1808
	mean ± SEM	
Age, years	50.0 ± 0.4	54.0 ± 0.3
Age categories	% (n)	
*18–39 years*	27.9 (433)	18.1 (322)
*20–39 years*	27.5 (427)	18.1 (321)
*40–59 years*	42.7 (664)	44.9 (797)
*60–79 years*	27.2 (422)	33.5 (595)
*≥ 80 years*	2.3 (35)	3.4 (61)
Sex, female (%)	69.0(1073)	67.6 (1222)
Education, completed College/University	39.9 (619)	38.1 (687)
Employment status		
*Employed full-time*	42.1 (655)	39.8 (720)
*Employed part-time*	17.7 (275)	16.1 (291)
*Homemaker*	12.1 (188)	9.4 (170)
*Retired*	24.5 (380)	30.4 (549)
*Student*	3.8 (59)	1.8 (33)
*Temporarily unemployed*	4.5 (70)	5.1 (92)
*Volunteer*	7.7 (120)	5.2 (94)
Level of combined household income, above cut-off points^a^	84.8 (1223)	87.8 (1490)
Perceived health, excellent and very good	54.0 (838)	54.1 (978)
Sense of belonging to local community, strong	73.3 (1121)	77.1 (1380)
Fruit and vegetable intake, ≥ 5 servings/day^b^	49.5 (751)	46.9 (824)
Physical activity index, active^c^	36.7 (570)	22.4 (405)
High blood pressure	18.0 (279)	21.5 (387)
Use antihypertensive medication	16.8 (261)	19.5 (352)
Smoking status, daily	24.0 (254)	20.4 (246)

Data represent unweighted values

^a^Low income cut-offs are defined by Statistics Canada as income thresholds below which families devote a larger share of income to purchasing food, shelter and clothing than average families. Low income cut-offs were calculated for each participant based on family size and community of residence [75]

^b^One serving of fruits and vegetables was defined as a medium fruit or half a cup of fresh, frozen or canned vegetables [76, 77]

^c^Active was defined as a total average daily energy expenditure ≥ 3.0 kcal/kg/day [77]

A comparison sample for the physical measures was drawn from the 2007–2009 and 2009–2011 waves of the Canadian Health Measures Survey [11, 30]. This approach allowed the majority of research funds to be channeled into intervening within the four HAC communities, rather than into creating costly and somewhat artificial, matched comparison communities. Although we had intended to use all non-HAC communities within Alberta as a comparison, the Alberta-based sample was too small [28], and therefore data were compared to a national sample.

#### Ethical approval

The study was approved by the University of Alberta’s Research Ethics Board. Respondents to the telephone survey provided verbal consent to participate, while those who participated in physical measures also provided written, informed consent.

#### Physical measurements

A trained measurement technician collected all anthropometric (height, weight, waist and hip circumference) and BP measurements according to standardized protocols. To facilitate comparisons with the reference population, all clinic procedures were adapted from the clinic procedures protocol developed for the Canadian Health Measures Survey [31]. Prior to all measurements participants removed their footwear, bulky clothing (gowns were provided), belt, all objects from their pockets, and any hair ornaments, jewellery, buns and braids from the top of the head.

#### Blood pressure

BP was measured prior to completing any other physical measures or completing any questionnaires. SBP and DBP were measured in a seated position on the right arm of each participant following a 15 min rest period using an appropriately sized automated BP cuff (BpTRU Model 300, BPTRU Medical Devices, Coquitlam, British Columbia, Canada). Six BP measurements were taken. Mean BP values were calculated by discarding the first, and averaging the last five BP values. BP was classified as normal (SBP < 130 mmHg and/or DBP < 85 mmHg), high-normal (SBP = 130–139 mmHg and/or DBP = 85–89 mmHg), or stage 1 hypertension (SBP = 140–159 mmHg and/or DBP = 90–99 mmHg) based on standards from the fifth report of the Joint National Committee on Detection, Evaluation and Treatment of High Blood Pressure [32]. Data for individuals with SBP ≥ 160 mmHg and DBP ≥ 90 mmHg were not considered as a comparison sample was not available (Canadian Health Measures Survey data were considered unreliable due to small sample sizes [11, 30]) .

#### Weight and height

Weight (to the nearest 0.1 lb) was measured using digital scales (LifeSource UC-321 Precision Personal Health Scale, Auto Control Medical Inc., Millcreek, Ontario, Canada) that were calibrated daily prior to use. Participants were requested to stand in the centre of the scale, with weight evenly distributed on both feet during the measurement. Standing height was measured using a portable stadiometer with a vertical backboard and a moveable headboard (Seca 214 Road Rod Portable Stadiometer, Seca, Chino, California, USA). Participants were asked to stand tall with arms hanging at the sides, feet together with weight evenly distributed between them, and their heels, buttocks, back and head touching the vertical backboard of the stadiometer. The measurement technician aligned the participant’s head in the Frankfort Plane and asked the participant to take a deep breath and hold it while the measurement was taken and recorded to the nearest 0.1 cm.

#### Body mass index

BMI was calculated from measured height and weight and classified as: normal weight (18.5–24.9 kg/m^2^), overweight (25.0–29.9 kg/m^2^), obese class I (30.0–34.9 kg/m^2^), obese class II (35.0–39.9 kg/m^2^), and obese class III (≥40.0 kg/m^2^) [33].

#### Waist and hip circumference, waist-to-hip ratio

Waist circumference was measured over light clothing while participants stood erect in a relaxed manner with arms hanging loosely at the sides, and weight evenly distributed between both feet. The measurement (to the nearest 0.1 cm) was taken at the end of a normal expiration at the mid-point between the bottom of the rib cage and the top of the iliac crest using a measuring tape (QM 2000 Measure Mate, Quick Medical, Issaquah, Washington, USA). Hip circumference was measured in the same manner, but at the maximal circumference of the hips or buttocks region (whichever was larger), and above the gluteal fold using the same measuring tape.

### Statistical analyses

Descriptive statistics were calculated for the baseline and follow-up samples and compared using t-tests. Weighting and bootstrapping (with 300 iterations) of the phone survey databases were used to obtain accurate estimates of variances. A series of logistic regressions were performed to determine whether participants who provided physical measurements were similar to those who participated in the telephone survey. As the physical measures sub-sample differed from the telephone survey sample on community of residence, age category, smoking, BMI, physical activity level, fruit and vegetable consumption, and self-reported health, re-weighting and bootstrapping (with 300 iterations) of the physical measures data was required and adjustments were made based on the survey weights calculated. The samples were stratified by age, and change between pre- and post-intervention time points in each of the age categories was assessed for each outcome using two-tailed t-tests. A comparison sample for the physical measures was drawn from the 2007–2009 and 2009–2011 waves of the Canadian Health Measures Survey [11, 30]. Changes in parameters over time between intervention and comparison groups were compared using meta-analytic procedures given that the analyzed data were from two different samples. The net difference in the change in outcomes (calculated as change in the intervention communities minus change in the comparison group) represented the effect of the intervention. Continuous data are presented as mean ± SEM with 95 % confidence intervals, frequencies are presented as percentages. All analyses were performed using SPSS (version 19.0, IBM Corporation, Armonk, NY, 2010), with *p <* 0.05 indicating statistical significance.

## Results

### Response rates and participant characteristics

The total number of phone numbers in the sample in all four HAC communities was 45212 in 2006 and 53785 in 2009. After excluding homes where the phone was not answered, a total of 12659 (27.9 %) and 8767 (16.3 %) people were asked to participate in the survey in 2006 and 2009, respectively. Of these, 4761 (2006) and 4733 (2009) individuals were eligible and completed the telephone interview. This represents an overall response rate of 10.5 % in 2006 and 8.8 % in 2009, with 37.6 % (2006) and 53.9 % (2009) of persons asked to complete the survey doing so. Of those who completed the telephone interview, valid physical measurement data were obtained from 1554 adults in 2006 and 1808 in 2009 (32.6 % and 38.2 % of those who completed Phase 1 interviews in 2006 and 2009, respectively). Characteristics of individuals from intervention communities who completed physical measurements are presented in Table 1. Socio-demographic characteristics of individuals within the comparison group were not publicly available and are therefore not reported.

### Blood pressure

**Fig. 1:**
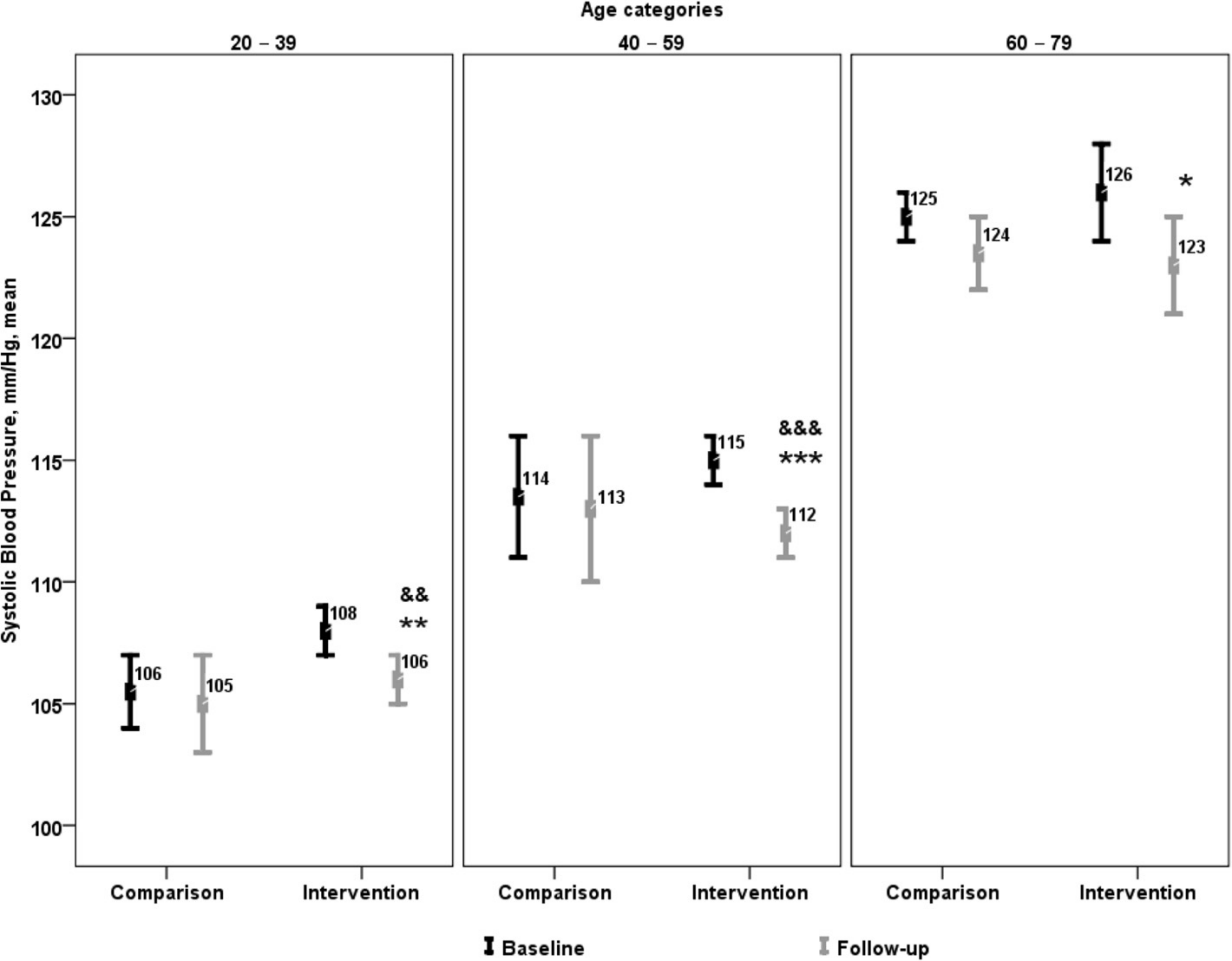
Change in mean systolic blood pressure within and between intervention and comparison groups. *p < 0.05 between follow-up and baseline for intervention communities. **p < 0.01 between follow-up and baseline for intervention communities. ***p < 0.001 between follow-up and baseline for intervention communities. ^&&^ *p <* 0.01 between follow-up and baseline changes in intervention and comparison groups. ^&&&^ *p <* 0.001 between follow-up and baseline changes in intervention and comparison groups

**Fig. 2:**
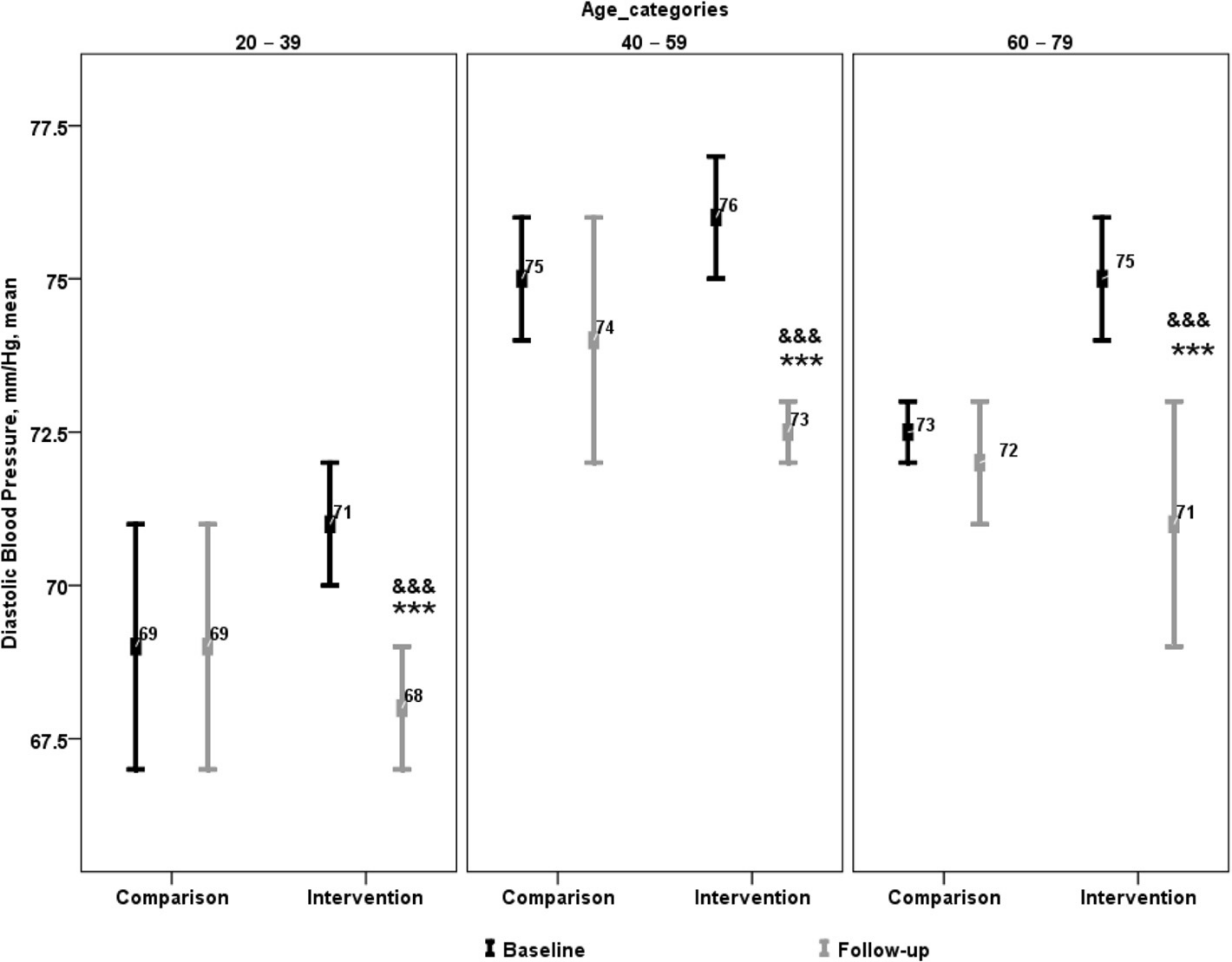
Change in diastolic blood pressure within and between intervention and comparison groups. ***p < 0.001 between follow-up and baseline for intervention communities. ^&&&^ *p <* 0.001 between follow-up and baseline changes in intervention and comparison groups

Changes in BP within intervention and comparison groups from 2006–2009 are presented in Table 2. At baseline, mean SBP and DBP values were higher in intervention communities than in the comparison group. There was a significant decline in the net adjusted SBP and DBP within most age groups in HAC communities from baseline to follow-up. The net decline in SBP was 1 mmHg in 20-39 year olds (*p=*0.006) and 2 mmHg in 40–59 year olds (*p =* 0.001) (Fig. 1), while the net decline in DBP was 3 mmHg in 20–39 year olds (*p <* 0.001), 2 mmHg in 40–59 year olds (*p <* 0.001) and 3 mmHg in 60–79 year olds (*p <* 0.001; Fig. 2). As a result, at follow-up mean SBP and DBP within intervention communities was equal to, or lower than values in the comparison group for all age categories, with the exception of SBP values within the 20–39 year old age group.

**Table 2:** Change in physical measures in intervention (HAC_2006_ and HAC_2009_) and comparison (CHMS_2007-09_ and CHMS_2009-11_) groups

Parameter	Age categories	Intervention^a^							Comparison^b^							Differential significance between changes in intervention and comparison groups *p*-value
		Baseline (*n* ***=*** 1554)			Follow-up (*n* ***=*** 1808)			∆	Baseline^c^(*n* ***=*** 3725)			Follow-up^d^(*n =* 3873)			∆	
		Mean	*95 % Confidence interval*		Mean	*95 % Confidence interval*			Mean	*95 % Confidence interval*		Mean	*95 % Confidence interval*			
			from	to		from	to			from	to		from	to		
Mean systolic blood pressure (mm Hg)	20–39	108	*107*	*109*	106	*105*	*107*	−2**	106	*104*	*107*	105	*103*	*107*	−1	0.006
	40–59	115	*114*	*116*	112	*111*	*113*	−3***	114	*112*	*116*	113	*111*	*116*	−1	0.001
	60–79	126	*124*	*128*	123	*121*	*125*	−3*	125	*124*	*126*	124	*122*	*125*	−1	0.087
Mean diastolic blood pressure (mm Hg)	20–39	71	*70*	*72*	68	*68*	*69*	−3***	69	*68*	*71*	69	*67*	*70*	0	<0.001
	40–59	76	*76*	*77*	73	*72*	*73*	−3***	75	*74*	*76*	74	*73*	*76*	−1	<0.001
	60–79	75	*74*	*76*	71	*70*	*73*	−4***	73	*72*	*73*	72	*71*	*73*	−1	<0.001
Normal blood pressure^e^ (%)	18–39	90.6	*88.2*	*92.9*	93.9	*92.1*	*95.7*	3.3*	95.4	*93.8*	*96.9*	94.7	*89.4*	*97.4*	−0.7	0.029
	40–59	76.3	*72.9*	*79.7*	85.2	*82.5*	*87.9*	8.9***	82.4	*78.8*	*85.9*	82.7	*77.4*	*86.9*	0.3	<0.001
	60–79	60.3	*54.5*	*66.1*	63.9	*58.6*	*69.2*	3.6	66.7	*64.2*	*69.1*	67.1	*62.9*	*71.0*	−0.6	0.403
	18–79	79.1	*77.0*	*81.1*	84.7	*83.0*	*86.5*	5.6***	84.4	*82.8*	*86.0*	84.1	*80.7*	*87.0*	−0.3	<0.001
High-normal blood pressure^e^ (%)	18–39	5.2	*3.4*	*7.0*	5.2	*3.5*	*6.8*	0	3.9	*2.6*	*5.1*	n/a	*…*	*…*	…	…
	40–59	12.2	*9.5*	*14.8*	11.0	*8.6*	*13.4*	−1.2	11.3	*8.7*	*13.9*	9.3	*6.8*	*12.6*	−2.0	0.772
	60–79	16.6	*12.2*	*21.0*	19.7	*15.3*	*24.1*	3.1	15.7	*12.8*	*18.6*	17.9	*14.5*	*21.9*	2.2	0.466
	18–79	10.2	*8.6*	*11.7*	10.2	*8.8*	*11.7*	0	9.2	*8.0*	*10.4*	8.9	*7.3*	*10.9*	−0.3	0.895
Stage 1 hypertension^e^ (%)	18–39	3.7	*2.2*	*5.2*	0.6	*0*	*1.1*	−3.1***	n/a	*…*	*…*	n/a	…	…	…	…
	40–59	8.8	*6.5*	*11.1*	2.7	*1.5*	*.0*	−6.1***	5.3	*4.0*	*6.6*	6.5^f^	*3.9*	*10.6*	1.2	<0.001
	60–79	16.7	*12.3*	*21.2*	13.9	*10.1*	*17.8*	−2.8	13.8	*11.6*	*15.9*	10.5	*8.2*	*13.5*	−3.3	0.638
	18–79	8.2	*6.8*	*9.7*	4.0	*3.0*	*4.9*	−4.2***	5.1	*4.5*	*5.8*	5.4	*3.8*	*7.5*	0.3	<0.001
Mean body weight (kg)	20–39	77.5	*75.9*	*79.2*	79.4	*77.8*	*81.0*	1.9	76.0	*74.2*	*77.7*	75.6	*72.5*	*78.7*	−0.4	0.111
	40–59	82.4	*80.9*	*83.9*	84.0	*82.4*	*85.6*	1.6	79.2	*77.4*	*80.9*	79.8	*77.7*	*81.8*	0.6	0.231
	60–79	81.9	*79.7*	*84.1*	82.9	*79.7*	*84.1*	1.0	77.5	*76.4*	*78.6*	78.0	*76.0*	*80.1*	0.5	0.585
Mean BMI(kg/m^2^)	20–39	26.69	*26.17*	*27.21*	26.68	*26.23*	*27.13*	−0.01	26.20	*25.64*	*26.77*	25.87	*25.09*	*26.65*	−0.33	0.848
	40–59	28.56	*28.09*	*29.04*	28.79	*28.32*	*29.26*	0.23	27.69	*27.18*	*28.19*	28.02	*27.38*	*28.66*	0.33	0.711
	60–79	29.24	*28.60*	*29.88*	29.62	*29.00*	*30.23*	0.38	28.23	*27.85*	*28.62*	28.43	*27.75*	*29.12*	0.20	0.486
Normal weight^g^ (%)	18–39	44.7	*40.7*	*48.7*	43.2	*39.5*	*46.9*	−1.5	48.2	*42.8*	*53.7*	49.7	*43.3*	*56.2*	1.5	0.532
	40–59	29.6	*25.9*	*33.3*	28.7	*25.2*	*32.1*	−0.9	32.7	*28.0*	*37.4*	32.2	*27.2*	*37.6*	−0.5	0.777
	60–79	20.4	*15.6*	*25.1*	20.6	*16.1*	*25.0*	0.2	27.9	*22.5*	*33.3*	25.6	*19.9*	*32.3*	−2.3	0.850
	18–79	34.0	*31.5*	*36.4*	33.1	*30.9*	*35.4*	−0.8	37.9	*33.3*	*42.4*	37.6	*33.1*	*42.3*	−0.3	0.674
Overweight^g^(%)	18–39	32.3	*28.5*	*36.1*	32.0	*28.5*	*35.4*	−0.4	29.7	*25.4*	*33.9*	28.4	*24.0*	*33.3*	−1.3	0.978
	40–59	35.9	*32.0*	*39.8*	36.5	*32.8*	*40.2*	0.6	41.4	*38.0*	*44.8*	37.4	*31.1*	*44.1*	−4.0	0.573
	60–79	40.2	*34.4*	*46.0*	35.8	*30.5*	*41.0*	−4.4	41.2	*37.2*	*45.3*	38.8	*34.0*	*43.9*	−2.4	0.368
	18–79	35.3	*32.8*	*37.7*	34.5	*32.2*	*36.8*	−0.8	36.7	*33.7*	*39.7*	34.2	*30.9*	*37.7*	−2.5	0.906
Obese, class I^g^ (%)	18–39	11.4	*8.8*	*13.9*	13.5	*11.0*	*16.1*	2.1	11.3	*9.8*	*12.7*	11.3	*8.5*	*14.9*	0	0.286
	40–59	20.6	*17.4*	*23.9*	21.4	*18.3*	*24.5*	0.8	15.9	*12.8*	*19.0*	17.3	*14.1*	*21.0*	1.4	0.897
	60–79	26.4	*21.2*	*31.6*	27.2	*22.3*	*32.1*	0.8	21.0	*16.1*	*25.8*	23.1	*20.4*	*26.1*	2.1	0.968
	18–79	18.0	*16.0*	*19.9*	19.3	*17.4*	*21.2*	1.3	15.1	*13.3*	*16.9*	16.2	*14.0*	*18.7*	1.1	0.646
Obese, class II^g^ (%)	18–39	5.5	*3.6*	*7.3*	6.0	*4.2*	*7.7*	0.5	5.0	*3.4*	*6.5*	4.8^f^	*2.8*	*8.1*	−0.2	0.686
	40–59	9.0	*6.7*	*11.4*	7.3	*5.3*	*9.3*	−1.7	6.3^f^	*4.2*	*8.5*	8.1^f^	*5.2*	*12.5*	1.8	0.189
	60–79	8.1	*4.9*	*11.4*	11.7	*8.1*	*15.2*	3.6	6.5	*4.5*	*8.5*	5.9	*4.5*	*7.8*	−0.6	0.126
	18–79	7.4	*6.1*	*8.8*	7.6	*6.3*	*8.8*	0.2	5.8	*4.7*	*7.0*	6.3	*4.6*	*8.7*	0.5	0.985
Obese, class III^g^ (%)	18–39	3.5	*2.0*	*5.0*	2.4	*1.2*	*3.5*	−1.1	2.8^f^	*1.6*	*4.0*	2.7^f^	*1.5*	*4.6*	−0.1	0.286
	40–59	4.3	*2.7*	*6.0*	5.8	*4.0*	*7.6*	1.5	3.2	*2.2*	*4.2*	4.2^f^	*2.8*	*6.1*	1.0	0.427
	60–79	4.5	*2.1*	*7.0*	4.2	*2.0*	*6.5*	−0.3	3.0^f^	*1.9*	*4.1*	4.6^f^	*2.8*	*7.5*	1.6	0.645
	18–79	4.0	*3.0*	*5.1*	4.1	*3.2*	*5.1*	0.1	3.0	*2.3*	*3.7*	3.7	*2.8*	*4.8*	0.7	0.671
Mean waist circumference (cm)	20–39	86.6	*85.2*	*88.0*	88.4	*87.3*	*89.6*	1.8*	86.9	*85.5*	*88.4*	85.7	*83.6*	*87.7*	−1.2	<0.001
	40–59	93.7	*92.4*	*94.9*	95.6	*94.4*	*96.9*	1.9*	93.4	*91.9*	*94.9*	93.8	*92.1*	*95.6*	0.4	0.062
	60–79	97.9	*96.1*	*99.8*	99.3	*97.6*	*101.0*	1.4	97.6	*96.2*	*98.9*	97.3	*95.4*	*99.1*	−0.3	0.255
Mean hip circumference (cm)	20–39	103.6	*102.6*	*104.6*	105.0	*104.1*	*105.9*	1.4*	102.7	*101.5*	*103.8*	102.1	*100.5*	*103.7*	−0.6	0.030
	40–59	105.6	*104.7*	*106.6*	106.7	*105.8*	*107.7*	1.1	104.3	*103.3*	*105.3*	104.5	*103.1*	*105.8*	0.2	0.139
	60–79	107.2	*105.9*	*108.6*	108.0	*106.8*	*109.3*	0.8	105.1	*104.3*	*105.9*	105.1	*103.8*	*106.4*	0	0.402
Mean waist/hip ratio	20–39	0.83	*0.82*	*0.84*	0.84	*0.83*	*0.84*	0.01	0.84	*0.84*	*0.85*	0.86	*0.85*	*0.86*	0.02***	0.807
	40–59	0.88	*0.88*	*0.89*	0.89	*0.89*	*0.90*	0.01	0.89	*0.89*	*0.90*	0.91	*0.90*	*0.92*	0.02***	0.518
	60–79	0.91	*0.90*	*0.92*	0.92	*0.91*	*0.93*	0.01	0.93	*0.92*	*0.94*	0.93	*0.93*	*0.94*	0	0.206

*CHMS* Canadian Health Measures Survey; HAC: Healthy Alberta Communities

^a^Values were adjusted for age, community of residence, smoking status, physical activity level, fruit and vegetable consumption, self-reported health, and self-reported BMI

^b^Total household population aged 18 to 79, except those meeting the CHMS exclusion criteria [78]

^c^Values are from the CHMS cycle 1 data tables [30]

^d^Values are from the CHMS cycle 2 data tables [11]

^e^Blood pressure (BP) was classified as normal (systolic BP < 130 mmHg and/or diastolic BP < 85 mmHg), high-normal (systolic BP = 130–139 mmHg and/or diastolic BP = 85–89 mmHg), or stage 1 hypertension (systolic BP = 140–159 mmHg and/or diastolic BP = 90–99 mmHg) [32]

^f^Use with caution, data with a coefficient of variation from 16.6 % to 33.3 % [11, 30]

^g^BMI was calculated from measured height and weight and classified as: normal weight (18.5–24.9 kg/m^2^), overweight (25.0–29.9 kg/m^2^), obese class I (30.0–34.9 kg/m^2^), obese class II (35.0–39.9 kg/m^2^), and obese class III (≥40.0 kg/m^2^) [33]

^n/a^Too unreliable to be published, data with a coefficient of variation >33.3 %, suppressed due to extreme sampling variability [11, 30]

*p < 0.05 between follow-up and baseline; **p < 0.01 between follow-up and baseline; ***p < 0.001 between follow-up and baseline

**Fig. 3:**
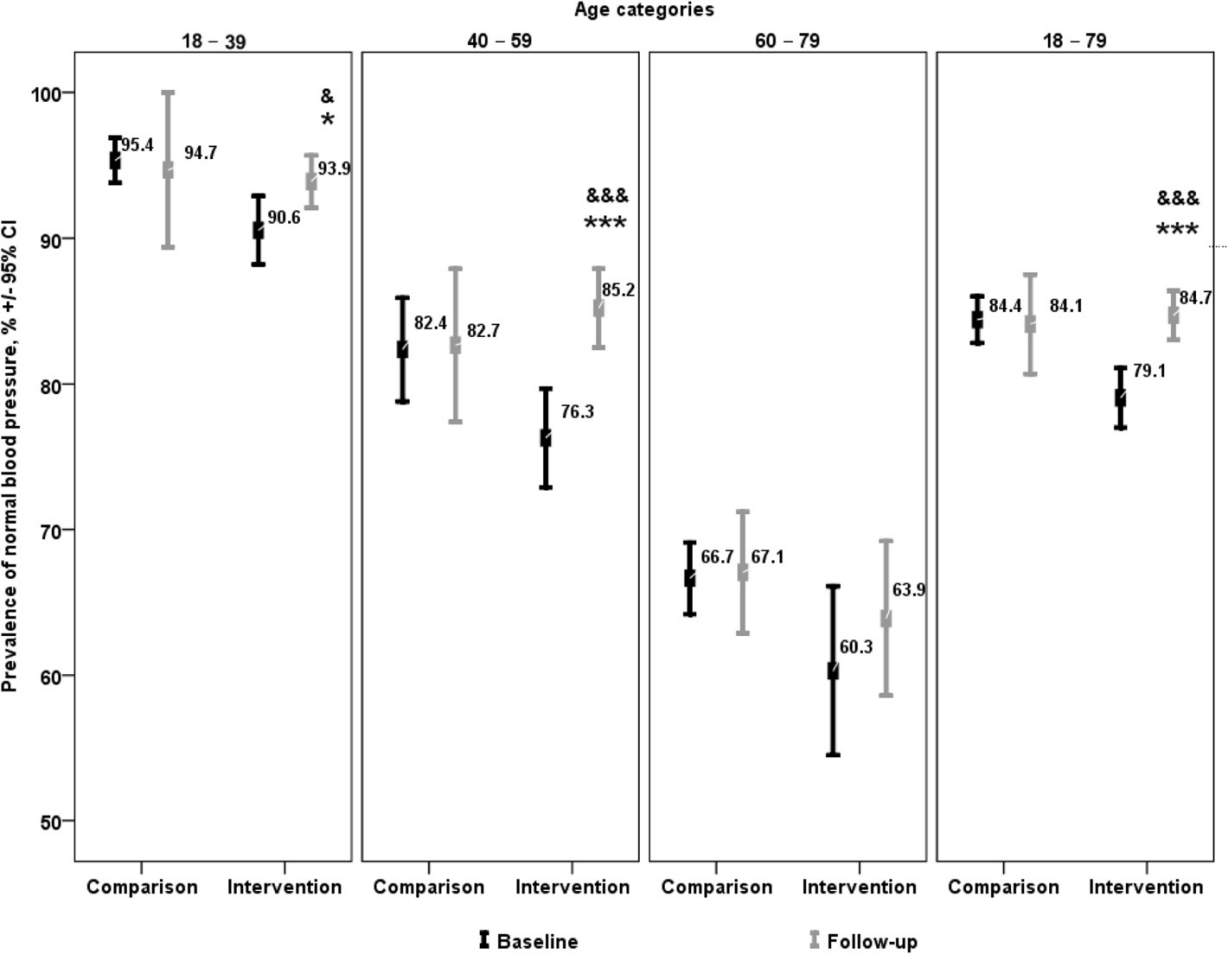
Prevalence of normal blood pressure in intervention and comparison groups. Normal blood pressure was defined as systolic blood pressure < 130 mmHg and/or diastolic blood pressure < 85 mmHg [32]. *p < 0.05 between follow-up and baseline for intervention communities. ***p < 0.001 between follow-up and baseline for intervention communities. ^&^ *p <* 0.05 between follow-up and baseline changes in intervention and comparison groups. ^&&&^ *p <* 0.001 between follow-up and baseline changes in intervention and comparison groups

**Fig. 4:**
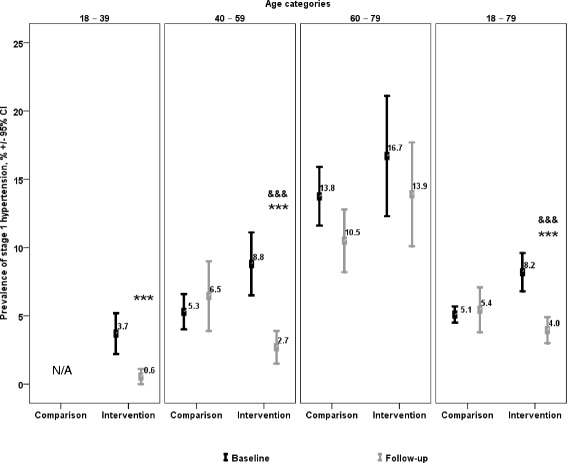
Prevalence of stage 1 hypertension in intervention and comparison groups. Stage 1 hypertension was defined as systolic blood pressure 140–159 mmHg and/or diastolic blood pressure 90–99 mmHg [32]. ***p < 0.001 between follow-up and baseline in intervention communities. ^&&&^ *p <* 0.001 between follow-up and baseline changes in intervention and comparison groups. n/a = too unreliable to be published, data with a coefficient of variation >33.3 %, suppressed due to extreme sampling variability [11, 30]

The proportion of individuals with normal BP increased significantly in intervention communities in all age groups except the 60–79 year old group (Fig. 3). No significant changes were observed within the comparison group. Changes within intervention communities were significantly greater than those in the comparison group overall (*p =* 0.029), and within the 18–39 (*p <* 0.001) and 40–59 year old (*p <* 0.001) age categories. A corresponding reduction in the proportion of individuals within intervention communities with stage 1 hypertension was also observed in all age groups except the 60–79 year old group (Fig. 4). By contrast, values in the comparison group were unchanged. The changes observed in intervention communities were significantly greater than those in the comparison group overall (*p <* 0.001), and for the 40–59 year old (*p <* 0.001) age categories. The outcome of these shifts was a net increase in the proportion of normotensive individuals of 4.0 % in the 18–39 year old group (*p =* 0.029), of 8.6 % in the 40–59 year old group (*p <* 0.001), and 5.9 % overall (*p <* 0.001), along with a net decrease in the proportion of stage 1 hypertensive individuals by 7.3 % among 40–59 year olds (*p <* 0.001) and 4.5 % overall (*p <* 0.001).

### Anthropometrics

There were no significant differences in body weight, BMI or in the distribution of BMI from 2006–2009 within or between the intervention and comparison groups (Table 2). Waist circumference increased significantly among 20–39 and 40–59 year olds in intervention communities (*p <* 0.05), while hip circumferences increased significantly among 20–39 year olds (*p <* 0.05) in intervention communities. Waist and hip circumference decreased, though not significantly, among 20–39 year olds in the comparison group. As a result, the net increase in waist circumference was 3.0 cm (*p <* 0.001) and 2.0 cm (*p =* 0.03) in hip circumference among 20–39 year olds in intervention communities.

The waist-to-hip ratio of 20–39 (*p <* 0.001) and 40–59 year olds (*p <* 0.001) in the comparison group increased significantly (Table 2). Waist-to-hip ratio remained unchanged within intervention communities and the magnitude of change did not differ between the intervention and comparison group.

## Discussion

A growing body of evidence indicates that unhealthy environments, including ready availability, affordability and convenience of energy-dense, nutrient-poor foods, and limited opportunities for physical activity are major drivers of unhealthy lifestyle behaviours and of attendant chronic disease and obesity in Canada and globally [2, 15]. HAC was a community-based intervention that sought to reduce risk factors for chronic disease and obesity through leveraging the collective capacities of communities, academics and policy makers to tackle these risk drivers. Study findings suggest that HAC-associated initiatives positively impacted SBP and DBP and reduced the overall prevalence of stage 1 hypertension across the four HAC communities in comparison with national trends. By contrast, anthropometric indicators remained largely unchanged, and in some cases worsened in intervention communities.

As one of the leading risk factors for stroke and ischaemic heart disease [6], and the leading risk factor for global disability and death [9], reducing the population prevalence of hypertension is an important public health goal. Findings from this study demonstrate that significant population-wide reductions in BP can be achieved through relatively diffuse, community-led interventions. The net reductions (change in intervention communities minus change in the comparison group) in SBP observed ranged from 1 to 2 mmHg, and from 2 to 3 mmHg for DBP across the various age groups. BP reductions of this magnitude can have positive health impacts, as a 2 mmHg reduction in SBP can reduce mortality from stroke by 10 % and mortality from ischaemic heart disease by 7 % [5], while lowering DBP by 5 mmHg can reduce the risk of stroke by 34 % and ischaemic heart disease by 21 % [34]. As a result of these changes the population distribution of BP shifted to the left, with a net increase in the proportion of normotensive individuals of 5.9 %, and a net decrease in the proportion of stage 1 hypertensive individuals of 4.5 %. These findings are encouraging in light of persistently high rates of hypertension in Canada and globally [35, 36].

The mechanism underlying the BP reductions is unclear; however, given the prominent role of lifestyle-related factors in BP homeostasis [13, 14], the targeting of these risk factors through HAC initiatives, and the stability in the proportion of participants in HAC communities taking anti-hypertensive medications throughout the study, changes in lifestyle-related behaviours are likely implicated. Indeed, others have shown that antihypertensive medications contributed to less than 25 % of the decline in SBP observed in England between 1994 and 2009 [37]. Lifestyle modifications that effectively lower BP include weight loss, reduced salt intake, consumption of a DASH-style (Dietary Approaches to Stop Hypertension) dietary pattern, increased potassium intake, moderation of alcohol intake and increased physical activity [13, 14]. Physical activity levels declined among individuals in HAC communities, while BMI and fruit and vegetable intake were unchanged [28], suggesting that a reduced sodium intake may have been a primary mechanism. A meta-analysis of short-term sodium reduction trials demonstrated a dose-responsive relationship between salt and BP, with a 1 g/d decline in salt intake leading to a 1 mmHg fall in SBP [38]. Although reduced alcohol consumption and/or adoption of a DASH-style dietary pattern might also be implicated, substantial changes in these areas are more likely to have been accompanied by weight loss. Notably, although alcohol intake increased in Finland between 1970 and 1997, BP declined during this period [39].

It is challenging to compare results across community-based interventions given their unique contexts and intervention strategies. Nevertheless, it is noteworthy that a number of other community-based interventions based on similar principles and strategies have also succeeded in reducing population-level BP. Between 1972 and 2007 in North Karelia, for instance, SBP declined by 11 mmHg in men and 19 mmHg in women, while DBP decreased by 9 mmHg in men and 14 mmHg in women (note that these values do not represent net reductions as a true comparison group was not available in the later years of the study) [21, 39]. These declines have been attributed to increased drug treatment and reductions in calorie, salt, and fat intakes [40–43].

In the Stanford Three Community Study, 2 years of extensive mass media campaigns resulted in a substantial and sustained reduction in BP within intervention communities, whereas the risk of cardiovascular disease based on composite risk factor indices increased within the comparison community over the study period [44]. The subsequent 5-year Stanford Five-City Project led to net reductions in BP ranging from 1.1 to 3.8 mmHg [45, 46]. In the former West Germany, a 7-year community-oriented cardiovascular disease prevention project found a net reduction in mean SBP and DBP of 2 %, as compared with the national trend [47], while a 5-year community-based intervention in the Maastricht region of the Netherlands also documented a significant reduction in SBP of 7.8 mmHg in men and 5.5 mmHg in women [48]. By contrast, no changes in population-level BP were observed in a number of other community-based interventions, including the Minnesota Heart Health Program [49], the Pawtucket Heart Health Program [50], the Women’s Lifestyle Hart Trial [51], the Kilkenny Health Project [52] and the Belgian Salt Intervention Trial [53].

One of the HAC’s primary goals was to reduce risk factors for obesity. Although BMI did not decline within intervention communities, it did remain stable over the course of the study, a positive finding from an obesity prevention perspective. However, BMI was also unchanged in the comparison population, and thus it is impossible to attribute this positive outcome to HAC-associated interventions. Waist and hip circumference increased in intervention communities within some age groups, a disturbing trend given their association with cardiovascular risk [54, 55]. Puzzling findings with respect to anthropometric parameters were also observed in the North Karelia project [41, 56, 57], the Stanford Five-City Project [58], China’s Beijing Fangshan Cardiovascular Prevention Program [59], and the Isfahan Health Heart Program in Iran [60]. Similarly, a meta-analysis of six community-wide interventions to prevent weight gain in children found only a very small reduction in BMI z-score (-0.09, CI -0.16 to -0.02) among children in intervention communities [61]. The well-known Fleurbaix-Laventie study, a community-based intervention to reduce childhood overweight and obesity in northern France, actually showed a non-significant trend toward an *increased* prevalence of overweight during the first 8 years of the study [62], a trend that was subsequently reversed in later years [63]. In the Be Active Eat Well program in Australia, large reductions in the prevalence of overweight/obesity in intervention and comparison communities appear to have occurred largely during the 3-year post-intervention period [64].

It is perhaps unsurprising that HAC failed to affect BMI, as HAC devoted substantial efforts to community capacity building and environmental change, an approach unlikely to affect population-level body weights over a 3-year period [22]. Whereas BP is influenced by a relatively discrete set of factors and is readily modifiable through pharmaceutical and lifestyle interventions [13, 38], body weight is influenced by a complex array of interacting factors and is highly resistant to change [65–67]. HAC-associated interventions may not have been of sufficient duration and intensity to change lifestyle behaviours to the extent required to produce significant and sustained community-wide energy deficits. Similarly, such interventions could not likely counter the powerful influence of existing food and physical activity environments that overwhelmingly promote unhealthy behaviours. Survey results support this interpretation, as physical activity levels declined, and fruit and vegetable intake was unchanged within HAC communities [28].

### Future studies

Disappointing results from several community-based interventions, along with the high cost of clinical risk factor assessment, has led to the suggestion that more realistic and proximal indicators of community change should be emphasized within community-based interventions [22, 68, 69]. As Sorensen and colleagues [22] have remarked, it is inappropriate to use clinical standards to judge the success of research conducted at a population-level. Our findings are in keeping with this recommendation. From the outset we recognized that traditional health outcomes-focused evaluation methods could not capture the value, context, and processes underlying this comprehensive initiative, and that change in clinical risk factors would be difficult to detect at a population-level within a 3-year time frame [27]. For this reason, we assessed multiple indicators of community-level change through evaluation of capacity building activities [27, 70], social network analysis, and assessment of environmental change (unpublished observations to be presented in a forthcoming publication), as we anticipated these proximal indicators would likely demonstrate change in advance of the more distal targets of health behaviours and outcomes. As expected, individual-level risk factors changed only minimally [28], whereas community environments (unpublished observations) and capacity for health-related change improved significantly [27, 70]. Rich contextual and process-related data also emerged [27, 70].

In our estimation, the success of HAC and other community-based interventions that prioritize environmental and policy change should be judged principally in terms of their success in doing so. While clinical outcomes remain important to assess (particularly over longer time frames) and are critical for mobilizing stakeholder support and driving political action [71], they should be regarded as secondary outcomes. Similarly, Johnston et al. [72] have remarked that outcome goals (such as obesity reduction targets) may be the wrong goals, as they fail to account for natural feedback mechanisms that act to resist weight loss, and can lead to unintended consequences whereby interventions may be deemed to have failed despite their potential to improve overall health. Process-related goals, by contrast, may prompt a deeper examination of environmental contexts and opportunities for change within them [72]. Thus, future community-based interventions could improve resource allocation by channeling more resources into interventions, and considering a truncated list of clinical indicators complemented by community-level indicators such as change in health-promoting environments and policies, and process measures that capture the complexity and richness of community change as it unfolds [18]. These more comprehensive data can also be valuable in attempts to link measurable change in environments with key behavioural and health outcomes, enabling identification of interventions that ‘tip’ communities in healthful directions [73].

### Strengths and limitations

HAC interventions were implemented in real-world settings, with all of their constraints and supports, providing policy makers and practitioners with community-tested evidence of impact and effectiveness. The physical measures data are highly reliable as they are based on objective assessments by trained measurement technicians. Comparison data were from a nationally representative sample to allow the majority of research funds to be dedicated to intervention activities, rather than creating costly and somewhat artificial comparison communities. Other community-based interventions have also taken this approach [71]. Nevertheless, this strategy was not without its limitations, as it precluded random assignment of communities to intervention and comparison conditions. The population-based sampling design enhances the generalizability of the present findings, however the use of repeated cross-sectional samples may have introduced bias due to sampling errors and the low response rate to the telephone survey recruitment strategy. Thus, it is not entirely clear whether the changes that were observed in BP in HAC communities are representative of the changes that would have occurred had the intervention been implemented in all Alberta communities. Socio-demographic characteristics of individuals within the comparison group were not available, and therefore we cannot assess the comparability of the intervention and comparison samples. Identification of appropriately matched comparison groups is always challenging within community-based interventions, however [74]. Follow-up data within HAC communities may be affected by in- and out-migration, introducing new residents with limited or no exposure to the interventions. The alternative approach of using a longitudinal cohort can be equally problematic, however, due to high drop-out rates which limit representativeness [18].

In previous reports, we have emphasized that building capacity for community health promotion is a process that requires a sustained, long-term investment of time and resources [70]. Although 3 years is a long duration relative to many other similar studies, it is a relatively short time frame within which to effect change in the well-entrenched lifestyle behaviours and social norms of entire communities. Indeed, Community Coordinators indicated the project ended before they could fully realize all of their objectives [70]. Thus, study findings represent early indicators of change, and community-wide health impacts may emerge over a longer time frame. HAC was a capacity building intervention, and therefore Community Coordinators worked to embed HAC-related activities within existing community structures to ensure their sustainability beyond the funding period [70]. Evidence suggests that some of the initiatives developed through the course of the study have been maintained [27], however it is unclear whether the observed reductions in BP have also been maintained.

## Conclusions

Population-level, community-based interventions aim to reduce the burden of disease through moderate risk reductions across large population segments, as even small shifts in the population distribution of health behaviours can have sizeable impacts on population-level health outcomes. The modest changes observed in this study, including an increase in the proportion of individuals in HAC communities with normal BP, and concurrent reduction in the proportion of individuals with stage 1 hypertension, suggest that HAC succeeded in shifting the population distribution of BP in a leftward direction. Notably, BP reductions were achieved in all age groups. If such reductions are maintained, reductions in cardiovascular disease might become apparent over time. By contrast, anthropometric indicators were unchanged and in some cases worsened within intervention communities, highlighting the enormous challenge of improving body weight in the context of pervasive obesogenic environments.

Communities are complex, dynamic entities consisting of individuals and organizations linked together by common physical, sociocultural, economic and political contexts. Producing change requires understanding how these diverse environments interact to shape individual and collective health-related behaviours, and developing interventions to effectively leverage them in health-promoting directions. The HAC experience highlights the complexity of this endeavour and shows that communities, in partnership with academics and government, can effectively mobilize to produce change in local environments on a scale sufficient to impact population-level health outcomes. However, whereas improvements in some clinical risk factors can be achieved through relatively diffuse and shorter-term community-level environmental change, improvements in others may require interventions of greater intensity and duration. To better capture the wide ranging benefits of community-based interventions the next generation of studies should measure proximal indicators of community-level environmental change, and should seek to understand the processes through which change occurs. Community-based interventions should not gauge their success merely in terms of their efficacy in changing shorter-term individual-level clinical health outcomes.

### Ethics and consent to participate

The study was approved by the University of Alberta’s Research Ethics Board. Respondents to the telephone survey provided verbal consent to participate, while those who participated in physical measures also provided written, informed consent.

### Availability of data and materials

Data supporting our findings are available in the manuscript.

## Abbreviations

BP: blood pressure
DBP: diastolic blood pressure
HAC: Healthy Alberta Communities
SBP: systolic blood pressure
